# Characterization and Antimicrobial Assessment of Cadmium Sulfide Nanoparticles

**DOI:** 10.3390/ijms27010432

**Published:** 2025-12-31

**Authors:** Ezinne Uchechi Ekwujuru, Moses Gbenga Peleyeju, Cornelius Ssemakalu, Mzimkhulu Monapathi, Michael Klink

**Affiliations:** Department of Natural Sciences, Faculty of Applied and Computer Sciences, Vaal University of Technology, Vanderbijlpark Campus, Vanderbijlpark 1911, South Africa

**Keywords:** antimicrobial resistance, bactericidal agents, nanoparticles, cadmium sulfide

## Abstract

Resistance to conventional antibiotics remains a global health challenge. The search for more effective antimicrobial agents has led to the consideration of nanoparticles due to their potential biocidal activities. This study synthesized, characterized, and evaluated the antimicrobial behavior of cadmium sulfide nanoparticles (CdS NPs) during incubations at 37 °C and at room temperature (rt; 23 to 27 °C). XRD results showed that the synthesized nanoparticles had a cubic zinc blende structure, while microscopic investigations confirmed the particle size to be 7.236 nm on average. UV-Vis spectroscopy showed that the nanoparticles are active in the visible light region. Raman spectroscopy results showed peaks at 302.3 cm^−1^ and 601 cm^−1^, which represent the first- and second-order longitudinal optical phonon. Agar well diffusion, minimum inhibitory concentration (MIC), and minimum bactericidal concentration (MBC) assays were conducted to investigate the antimicrobial activity of CdS NPs (50 mg/mL, 25 mg/mL, and 10 mg/mL) against *Escherichia coli* and *Staphylococcus aureus*. CdS NPs were effective against both test organisms. However, they were more effective against Gram-negative *E. coli*. The higher the concentration of CdS NPs, the more effective they were against the test organisms. Furthermore, MBC results showed greater bactericidal activity of CdS NPs at 37 °C. With increasing incidences of antimicrobial resistance against conventional antimicrobial agents, especially in wastewater treatment, nanoparticles are considered promising alternatives and the next generation of antimicrobial agents.

## 1. Introduction

The chase for alternative antimicrobial agents has increased over the years because of the alarming increase in bacterial resistance to traditional antimicrobial agents. This antimicrobial resistance has become a silent pandemic and is a challenge to global health [1]. Pathogenic organisms such as *Clostridium tetani*, *Escherichia coli*, *Pseudomonas aeruginosa*, and *Staphylococcus aureus* are known to cause diseases, especially in immunocompromised people [2]. Antimicrobial agents are used to limit or prevent the growth of microbes [3,4], but the overuse and misuse of these agents have led microorganisms to develop resistance, rendering many conventional treatments unsuccessful [5,6]. Genetic mutations and horizontal gene transfer allow microbes to acquire and share these resistance traits [7].

The problem is further compounded by the persistence of pathogenic microorganisms in aquatic ecosystems, including wastewater environments. If inadequately treated, these microbial contaminants end up in open surface water and pose a serious risk to aquatic biota and human health [8,9]. Unfortunately, conventional wastewater treatments are costly and technically limited in removing certain pathogenic organisms [10,11]. Chemical treatments with chlorine and ozone add to the contaminants by introducing toxic by-products, in addition to not completely removing microorganisms [12]. Physical methods such as filtration and sedimentation are also not effective [13]. Also, biofilms affect the efficacy of conventional antimicrobial agents [14]. These problems have prompted the need to explore other antimicrobial strategies that can serve as effective alternatives to control the growth of microbial populations [15]. Strategies based on nanotechnology have given hope in the control of microbes and resistant traits, thus protecting the environment and preserving human health.

Metal-based nanoparticles such as CdS nanoparticles (CdS NPs) have emerged as promising antimicrobial agents because of their unique mechanisms of action, which differ greatly from conventional antibiotics [15]. These mechanisms include the generation of reactive oxygen species (ROS), disruption of microbial cell membranes, and interactions with intracellular materials such as DNA, proteins, and lipids, which lead to cell death [16,17]. CdS NPs, through these mechanisms, have been noted to exhibit a broad spectrum of antimicrobial activity against both Gram-positive and Gram-negative bacteria, including some fungi [18,19]. The generation of ROS by CdS nanoparticles is a critical factor in their antimicrobial efficacy, as ROS can damage cellular components such as DNA, proteins, and lipids, ultimately resulting in cell death [20,21,22].

CdS NPs are easy to synthesize, highly stable, and exhibit good physico-chemical and structural properties [23]. Cadmium is a well-known toxic metal, but it demonstrates reduced toxicity when present as cadmium sulfide, which, in turn, contributes to its biocompatibility [24]. CdS NPs are utilized in some other applications, such as sensing [25,26], drug delivery [27], as pigments in plastics and paints [28], and as anticancer agents [27]. Several methods have been developed for the synthesis of CdS NPs, including wet chemical methods, microwave-assisted co-precipitation, microwave irradiation, reverse micellar techniques, electrochemical deposition, Langmuir–Blodgett methods, solvothermal and hydrothermal processes, green synthesis, gamma irradiation, biological approaches, and mechanochemical methods [29]. The synthesis methods of CdS NPs play a critical role in determining their size, shape, and surface characteristics, which affect their applications, biological interactions, and antimicrobial activity [30,31]. Among these methods, the wet chemical method is preferred and used in this work due to its high yield, cost-effectiveness, simplicity, time conservation, and flexibility [24].

Previous studies have extensively utilized a range of characterization techniques to examine the properties and structure of CdS NPs [32,33]. Such characterization methods include Fourier transform infrared (FTIR) spectroscopy, energy-dispersive X-ray (EDX), X-ray diffraction (XRD), scanning electron microscopy (SEM), transmission electron microscopy (TEM), UV-Visible spectroscopy, and Raman spectroscopy. These techniques offer an extensive understanding of the physical, chemical, and structural features of CdS NPs, which help in determining the potential application and optimization criteria.

Clean and safe water is a critical resource for a flourishing society and a thriving economy [34]. However, consuming contaminated water can result in waterborne diseases such as cholera, diarrhea, dysentery, hepatitis A, and typhoid fever [35]. Addressing these challenges aligns with the United Nations Sustainable Development Goals established in 2015, particularly SDG 3 (Good Health and Well-Being) and SDG 6 (Clean Water and Sanitation) [36]. In this context, nanomaterials have emerged as promising tools for improving water treatment technologies due to their high surface area and enhanced antimicrobial properties. Cadmium sulfide nanoparticles, in particular, have the potential to inactivate pathogenic microorganisms in aqueous environments. Their physicochemical properties suggest possible applicability in disinfection processes within water treatment systems. Such innovations will promote cost-effective removal of pathogens and reduction in disease spread [37]. The present study, therefore, aims to synthesize and characterize cadmium sulfide nanoparticles while evaluating their antimicrobial potential at different temperatures against potentially pathogenic bacterial species. Specifically, at 37 °C, which corresponds to physiological temperature and is often used in antimicrobial evaluation, and room temperature (23–27 °C), which represents environmental or ambient conditions. The purpose of conducting the tests at these two temperatures is to determine whether temperature variation affects the antimicrobial activity of CdS NPs. This comparison gives an understanding of the activity and potential use of CdS NPs both in biological systems and under normal environmental conditions. Also, understanding the temperature-dependent behavior of CdS NPs will help in optimizing their use in any disinfection application. To our knowledge, this is the first study of its kind.

## 2. Results

### 2.1. Characterization of Nanoparticles

#### 2.1.1. Fourier Transform Infrared (FTIR) Spectroscopy

**Figure 1:**
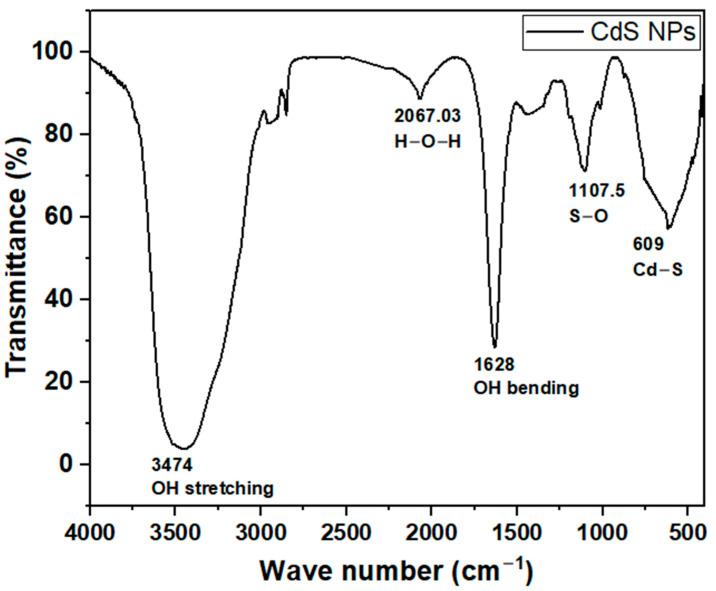
FTIR spectrum of CdS NPs.

To determine the surface functional groups of the synthesized cadmium sulfide nanoparticles, FTIR analysis was carried out, and the resulting spectrum is presented in Figure 1. The spectrum shows distinct peaks around 609, 1107.5, 1628, 2067, and 3474 cm^−1^. These peaks reveal the presence of various surface functional groups related to the synthesized CdS nanoparticles.

#### 2.1.2. The Raman Spectroscopy Analysis

**Figure 2:**
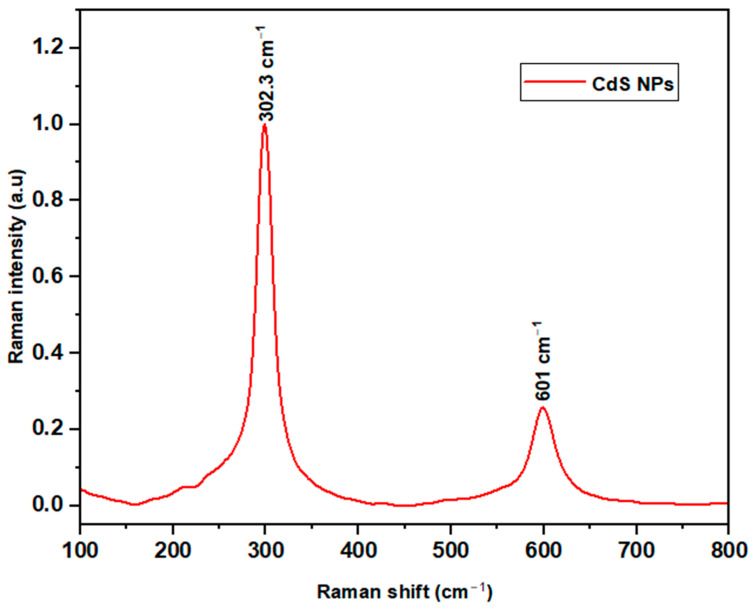
Raman spectrum of prepared CdS NPs.

The Raman spectrum (Figure 2) showed two peaks located at 302.3 cm^−1^ and 601 cm^−1^. These distinct peaks indicate vibrations that are characteristic of the nanomaterial. No additional peaks were observed within the scanned range.

#### 2.1.3. Structural and Surface Morphological Studies Using SEM

**Figure 3:**
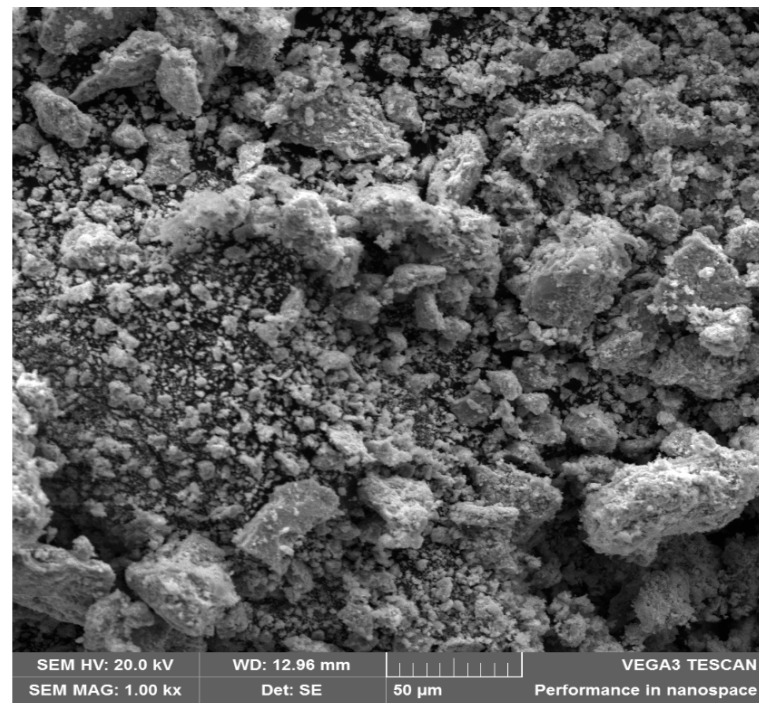
SEM image of CdS NPs.

The SEM image of the synthesized CdS NPs is presented in Figure 3. The image shows predominantly spherical, smooth-surfaced nanoparticles that are closely packed with slight agglomeration.

#### 2.1.4. Elemental Analysis Using EDX

**Figure 4:**
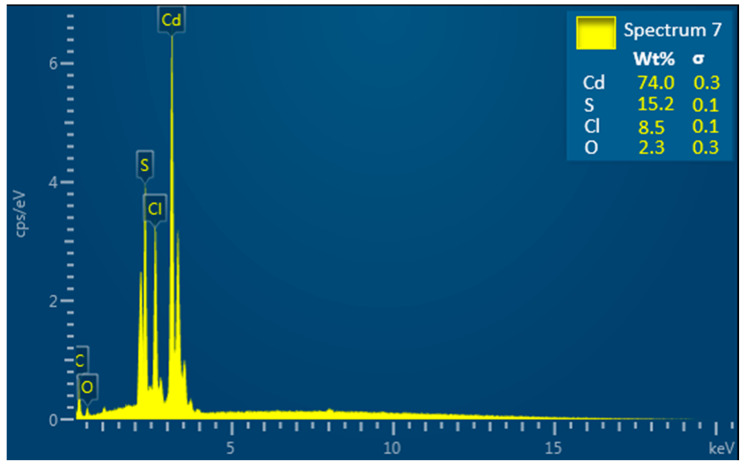
EDX spectrum of CdS nanoparticles.

The elemental composition and degree of purity of the synthesized CdS NPs were investigated using EDX. The EDX spectrum (Figure 4) shows the presence of cadmium, sulfur, Cl, O, and C.

#### 2.1.5. Structural and Surface Morphological Studies Using TEM

**Figure 5:**
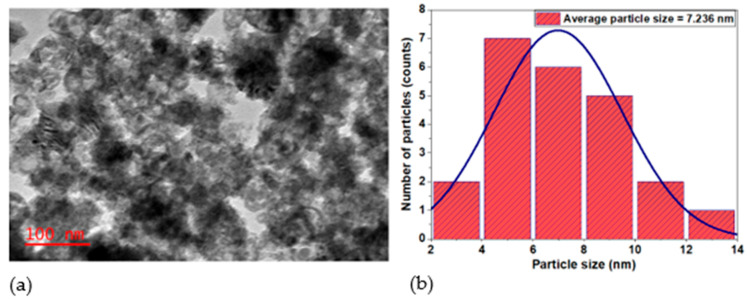
(**a**) TEM image of CdS NPs; (**b**) particle size distribution curve.

Figure 5a represents the TEM image of the CdS NPs recorded at a scale of 100 nm. The particles are aggregated and look spherical. The TEM image was analyzed using ImageJ software (version number 1.54k), and the average particle size was determined to be 7.236 nm. Figure 5b shows the histogram of the particle size distribution and the corresponding Gaussian fitting.

#### 2.1.6. Optical Studies Using Ultraviolet–Visible Spectrophotometer

**Figure 6:**
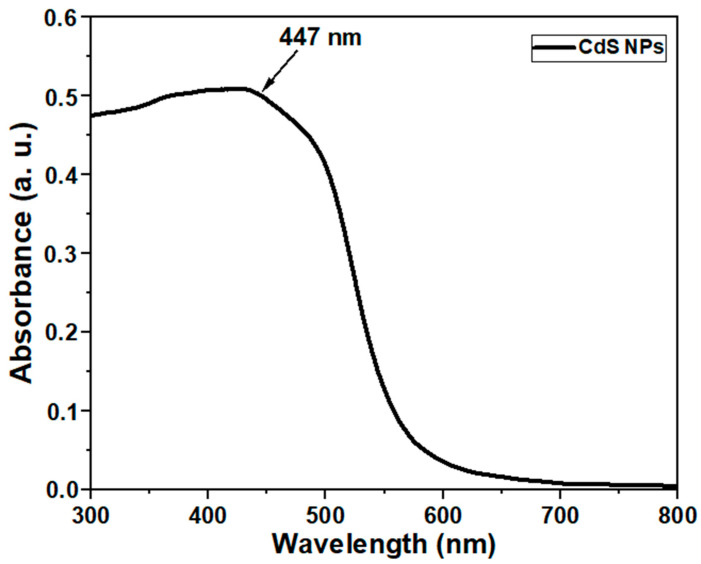
UV-vis absorption spectrum of CdS NPs.

The optical properties of the synthesized CdS NPs show the presence of broad peaks in the visible light region at around 447 nm, as shown in Figure 6.

#### 2.1.7. XRD Patterns

**Figure 7:**
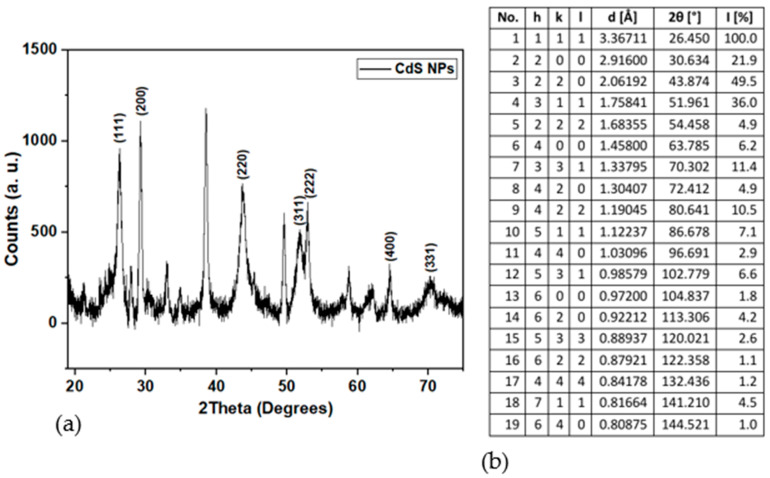
(**a**) XRD pattern of CdS NPs; (**b**) XRD standard card for CdS NPs.

The X-ray diffraction (XRD) pattern of the synthesized CdS NPs is presented in Figure 7a, and the standard card is displayed in Figure 7b. Multiple peaks were observed with notable ones associated with the synthesized nanoparticles at 2θ values of 26.29°, 29.22°, 43.67°, 51.85°, 54.10°, 64.02°, and 70.08°.

### 2.2. Evaluation of Antimicrobial Activity of CdS NPs

#### 2.2.1. Agar Well Diffusion Assay

**Figure 8:**
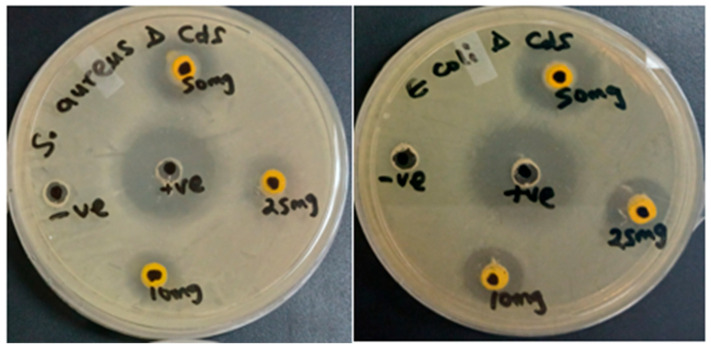
Indication of zones of inhibition on MH agar plates against *Staphylococcus aureus* and *Escherichia coli*.

Figure 8 shows varying antimicrobial activity of CdS NPs at varying concentrations (50 mg/mL, 25 mg/mL, and 10 mg/mL). The clear zones on the Mueller–Hinton (MH) agar plates show the degree of antibacterial activity of the nanoparticles. The diameters of inhibition zones in millimeters are displayed in Table 1, with Neomycin (positive control) having the largest zone of inhibition.

#### 2.2.2. Minimum Inhibitory Concentration (MIC)

**Figure 9:**
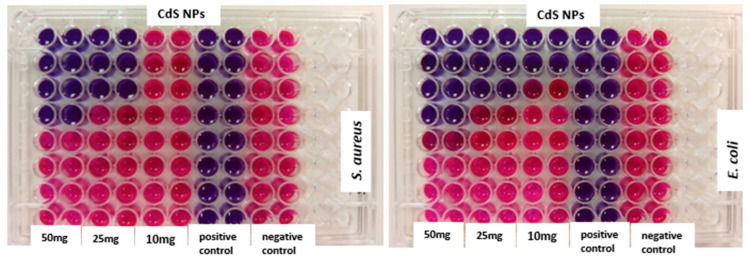
Minimum inhibitory concentrations of CdS NPs in *Staphylococcus aureus* and *Escherichia coli* at 37 °C. Blue color indicates ‘bacterial growth inhibition; pink color indicates ‘no bacterial growth inhibition’.

Figure 9 shows the MIC results for the synthesized CdS NPs at various concentrations (50 mg/mL, 25 mg/mL, and 10 mg/mL). Bacterial growth was inhibited by these concentrations of CdS NPs. The MIC values observed ranged from 25 to <0.391 μg/mL and are shown in Table 2.

#### 2.2.3. Minimum Bactericidal Concentration (MBC)

MBC results in Table 2 showed that CdS NPs are bactericidal at both room temperature and at 37 °C. Lower MBC values are observed at 37 °C and also against *E. coli*.

## 3. Discussion

FTIR spectrum analysis presented in Figure 1 shows peaks around 609 cm^−1^ indicating the presence of Cd-S stretching bonds, which is also observed in the literature [38,39]. The band around 1107.5 is assigned to stretching vibrations of the sulfate group, such as S-O [40,41], while the presence of water (H-O-H) is evidenced by its weak bending vibration at around 2067.32 cm^−1^ [42]. Peaks at 1628 and 3474 cm^−1^ represent O-H bending and stretching vibrations, respectively. Similar vibrational bands were also noted in other works [38,43].

The Raman spectrum in Figure 2 showed two peaks at 302.3 cm^−1^ and 601 cm^−1^. These are assigned to the first-order and the second-order longitudinal optical (LO) phonon vibrational modes, respectively, found in the synthesized CdS NPs. The prominent peak at the first-order longitudinal optical (1 LO) phonon is characteristic of CdS [44].

The SEM image of the synthesized nanomaterial is presented in Figure 3. The image shows predominantly spherical, smooth-surfaced nanoparticles, indicating a crystalline morphology. A certain level of agglomeration is also noted. The agglomeration may be due to the small particle size (<10 nm), the sample deposition method (a thin layer of the sample was introduced onto a carbon tape) before analysis, and the absence of a stabilizing agent [45]. These morphological characteristics are similar to those in other works [38,46]. The problem of aggregation can be overcome through sonication. Sonication disrupts agglomerates and homogenizes nanoparticle dispersion [47]. It also helps in increasing the specific surface area of the nanoparticles, thereby enhancing the contact with bacterial cells [21].

The EDX spectrum of CdS NPs in Figure 4 confirms the successful synthesis of CdS NPs with cadmium and sulfur as the major constituents. Minor peaks of Cl, O, and C are also present. Cl is a residue of the precursor material (CdCl_2_) used in the synthesis of the nanomaterial. O is a result of surface oxidation characteristic of CdS [48], and C is from the coating material used to improve the conductivity of the nanomaterial during analysis.

The TEM image of the synthesized CdS NPs (Figure 5a) shows aggregates of uniform, fine, and near-spherical crystalline-like particles and is consistent with the work of Dumbrava et al. [46]. The irregularity of the shape is due to the absence of a stabilizing agent, which made the nanoparticles clump together, resulting in aggregates of various shapes and sizes instead of remaining as well-defined spherical particles [45]. The average particle size was 7.236 nm, which was calculated from the TEM image using ImageJ software (version number 1.54k). The histogram in Figure 5b shows that most of the particle sizes were between 4 and 10 nm. The small particle sizes of the synthesized CdS NPs increase their effectiveness as an antimicrobial agent due to their large surface area, which effectively interacts with microbial cells [49]. The small particle size of CdS NPs synthesized in this study suggests quantum dot-sized nanostructures and may exhibit quantum confinement effects and associated fluorescent properties [50]. However, in view of the specific aims of this study, the investigation of fluorescence characteristics was considered beyond its scope and therefore not included.

The optical spectrum of the CdS NPs with broad peaks in the visible light region at around 447 nm (Figure 6) is characteristic of the nanomaterial and is consistent with those in the literature [32,39]. This correlates with the surface plasmon resonance, which indicates the presence of stable nanoparticles [32].

The XRD pattern in Figure 7a indicates that the prepared nanomaterial has a cubic crystal structure. Three peaks with 2θ values of 26.29°, 43.67°, and 51.85° correspond to the hkl planes of (111), (220), and (311), respectively [44,46]. These reflections are well-known signature indicators of the cubic CdS crystal. In addition, other hkl planes associated with cubic CdS crystals (200), (222), (400), and (331) located at 2θ values of 29.22°, 54.10°, 64.02°, and 70.08°, respectively, were also evident. The presence of these additional planes further validates the crystal ordering of the synthesized CdS NPs. The Joint Committee on Powder Diffraction Standards (JCPDS) card no. 01-075-1546, presented in Figure 7b, was used for peak identification. The unassigned diffraction peaks seen in the XRD spectra may be attributed to the instrumental effects [51,52] or trace impurities originating from the precursor material (CdCl_2_). This assumption is validated by the result of the EDX analysis (Figure 4), which shows the presence of Cl.

The average crystallite size of CdS NPs was determined using the Debye–Scherrer formula [44,46],

D = Kλ/βcosƟ(1)

where D—crystallite size; K—Scherrer’s constant (0.9); λ—wavelength of Cu Kα radiation (1.54060 Å); β—full width at half maximum (FWHM); and θ—Bragg angle. The average crystallite size was estimated to be 3.72 nm. This value is smaller than the particle size obtained from TEM analysis (7.236 nm). Such a difference is expected, due to the agglomeration of the particles observed in TEM (Figure 5a).

In the antimicrobial studies, the effect of the synthesized CdS NPs was studied at two temperatures: physiological temperature (37 °C) and room temperature (23 to 27 °C) to evaluate the impact of temperature on the antimicrobial efficacy of CdS nanoparticles at varying concentrations. The agar well diffusion assay was used to qualitatively screen CdS NPs for their antimicrobial activity against two test organisms at different concentrations (50 mg/mL, 25 mg/mL, and 10 mg/mL). Figure 8 shows the varying antimicrobial activity of CdS NPs. The clear zones formed on the Mueller–Hinton (MH) agar plates show the degree of antibacterial activity of the nanoparticles. The larger the inhibition zone, the more effective the CdS NPs are against the test organisms [53].

Table 1 displays the diameters of inhibition zones in millimeters at different temperatures. Neomycin (positive control) has the largest zone of inhibition, thus exhibits a higher growth inhibitory effect against the test organisms. A study by Regmi et al. [20] aligns with the present study, where high antimicrobial activity was observed for the commercial antibiotic (tetracycline) than for CdS NPs.

Larger zones of inhibition by CdS NPs were observed at room temperature (23 to 27 °C) (Table 1). According to studies by Tuttle et al. and Xie et al., 37 °C is the optimal temperature for the growth of *E. coli* and *S. aureus* [54,55]. Thus, room temperature is not ideal for the proper growth of these test microorganisms [56]. At lower temperatures, microbial enzymatic processes are slowed, resulting in reduced growth [57]. Consequently, the nanomaterial was more effective in inhibiting the growth of the microbes at room temperature. Also, CdS NPs were more effective against *E. coli* than *S. aureus* under room temperature incubation. The agar well diffusion assay results show that the nanomaterial responds more effectively under ambient conditions and displays stronger activity against Gram-negative bacteria (*E. coli*) than against Gram-positive bacteria (*S. aureus*).

Antimicrobial activity of CdS NPs was found to be concentration-dependent. Higher antimicrobial activity was observed at the highest concentration (50 mg/mL) on both test organisms (Table 1). These findings show that the increase in concentration of the nanoparticles also enhances their antimicrobial activity. According to Regmi et al. [41], an increase in the concentration of nanomaterials increases the quantity of the nanomaterial. This results in greater interaction with the test organism and enhanced antimicrobial activity.

Minimum inhibitory concentration (MIC) was also used to assess the antimicrobial efficacy of the nanomaterial against the test organisms. It is a quantitative method and is defined as the lowest concentration in µg/mL of an antimicrobial that, under strictly controlled in vitro conditions, completely stops visible growth of the test strain of an organism [53,58]. In this study, non-fluorescent resazurin dye (blue color) was used as the redox indicator for the assay. In the presence of viable microbial cells, the dye was irreversibly reduced to the fluorescent resorufin (pink color) [59], while non-viable cells retained the blue color of the dye [59,60]. The concentration at which no color change occurred (blue color) was taken as the minimum inhibitory concentration.

Figure 9 shows the MIC results for the synthesized CdS NPs at various concentrations (50 mg/mL, 25 mg/mL, and 10 mg/mL). Bacterial growth was inhibited by varying concentrations of CdS NPs. The lower the MIC, the more effective the antimicrobial agents [53]. As shown in Table 2, MIC values observed ranged from 25 to <0.391 μg/mL with a lower MIC of 3.125 μg/mL for CdS NPs (50 mg/mL) against *Escherichia coli*. CdS NPs at 50 mg/mL were more effective than their counterparts, confirming the concentration dependence of the antimicrobial activity. The MIC values for the RT incubation are generally lower than those of the physiological incubation temperature (37 °C). This still indicates that RT is more favorable to the antimicrobial activity of CdS NPs. Overall, the behavior of the nanoparticles toward the test organisms at both incubation temperatures did not follow a consistent pattern in the MIC results. However, in some cases, the MIC values were lower for *E. coli* than for *S. aureus*, while in other cases, the two organisms exhibited the same MIC values. This observation further supports the higher susceptibility of *E. coli* to CdS NPs.

Meanwhile, the lowest MIC values (<0.391 μg/mL) were observed for Neomycin, indicating that the commercial antibiotic was more effective against the test organisms. These results agree with those of the agar well diffusion assay (Figure 8), where the largest zones of inhibition were observed for Neomycin. Notwithstanding the high performance of these conventional antibiotics, the increased resistance associated with commercial antimicrobial agents has prompted an urgent need to explore other alternatives [5].

Antimicrobial agents are generally classified as bacteriostatic or bactericidal agents [61,62]. Bacteriostatic agents stop (inhibit) the growth of bacterial cells without killing them [61,63]. On the other hand, bactericidal agents kill (eliminate) bacterial cells. Relevant to MIC, nanoparticles with an MBC value below MIC are referred to as bactericidal [64]. Conversely, nanoparticles with MBC above MIC are referred to as bacteriostatic [65]. MBC results in Table 2 show that CdS NPs were more bactericidal at 37 °C than at RT incubation, as evidenced by the lower MBC values. This result contradicts the Agar well and MIC assay results (Table 1 and Table 2), where RT incubation outperformed the 37 °C incubation. This result indicates that the CdS NPs are more inhibitory (bacteriostatic) in their mode of action at RT incubation, while demonstrating a killing (bactericidal) effect at 37 °C. The results further demonstrate that the higher the concentration of CdS NPs, the more bactericidal. Furthermore, when compared to *S. aureus*, CdS NPs were more bactericidal against *E. coli*. Also, it is worthy to note that, RT incubation had no bactericidal effect on *S. aureus*.

Generally, it can be deduced that incubation temperature has a significant effect on the antimicrobial activity of CdS NPs, with 37 °C incubation exhibiting a more pronounced bactericidal effect, whereas room temperature (23 to 27 °C) produced mainly a bacteriostatic effect. The 37 °C incubation was able to exhibit a more bactericidal effect because at this ideal growth temperature, the microorganisms are vegetatively active with very active membrane transport [54,55]. Consequently, the uptake and accumulation of the nanoparticles are facilitated, which causes more severe cellular damage, leading to cell death compared to incubation at RT when the metabolic activity is slow [57].

Findings from agar well diffusion method and MIC and MBC assays show that CdS NPs demonstrated higher antimicrobial activity against *E. coli* than *S. aureus* at both incubation temperatures (37 °C and room temperature). This can be attributed to the structural differences in the cell wall of bacterial species [20]. The Gram-negative bacterium, *E. coli*, has a thin peptidoglycan layer, which allows for easy permeability of nanoparticles into the bacterial cell wall, disrupting intracellular components. On the other hand, *S. aureus*, a Gram-positive bacterium, has a thick peptidoglycan layer, which acts as a barrier and makes it difficult for CdS NPs to pass through the bacterial cell wall [32].

CdS NPs were able to exhibit their antimicrobial activity through the release of reactive oxygen species into the bacterial cell and its micro-environment [66]. High levels of ROS are capable of damaging bacterial cells by disrupting vital processes through carbonylation of proteins, DNA/RNA breakage, membrane structure destruction, and lipid peroxidation [20,21,22]. The damage to the bacterial cells cause influx of Cd^2+^ ions into the cells, thereby causing further toxicity. Cd^2+^ ions can react with the thiol group of proteins to produce ROS [32], mainly superoxide [22]. The superoxide can be dismutated to hydrogen peroxide, which then produces a very toxic hydroxyl radical that increases the lethality of the nanomaterials [22].

### 3.1. Limitation of the Study

A key limitation of this study is the absence of experiments involving real contaminated water samples or simulated water treatment systems. Although the antimicrobial activity of the synthesized CdS nanoparticles was demonstrated under controlled laboratory conditions, these tests do not account for the inherent physicochemical and biological complexity of aqueous environments, such as the presence of organic matter, interfering ions, and mixed microbial populations. Consequently, the findings in the current study should be regarded as preliminary, and further studies are required to evaluate the performance, stability, and safety of the nanoparticles under real water treatment conditions.

### 3.2. Future Perspectives

Future research may expand the scope of this work toward investigating the antimicrobial capability of CdS nanoparticles in real water samples, as well as their diagnostic and theranostic applications. Given their nanoscale dimensions, studies of their fluorescent properties could enable their use in bio-imaging and sensing platforms [67]. In addition, surface stabilization or functionalization with antimicrobial agents or dyes may improve their performance and biocompatibility [68]. Such modifications could enable dual functionality by combining antimicrobial and diagnostic capabilities.

## 4. Materials and Methods

### 4.1. Synthesis of Cadmium Sulfide Nanoparticles

CdS NPs were prepared using a wet chemical synthesis method [44]. Briefly, 0.1 mole each of CdCl_2_ and Na_2_S (Sigma-Aldrich, Johannesburg, South Africa) was dissolved separately in 25 mL of ultrapure water and stirred continuously until completely dissolved. The Na_2_S solution was added to the CdCl_2_ solution in a dropwise manner while stirring at 30 °C. The solution changed into a yellowish color, confirming the successful synthesis of CdS NPs [42,69]. The formed CdS NPs were collected by centrifugation followed by repeated washing (three times) with ethanol. The obtained product was dried at 60 °C and stored at 4 °C in a sealed container for subsequent use to prevent the deterioration of the nanoparticles [70].

### 4.2. Characterization of the Synthesized Cadmium Sulfide Nanoparticles

The general characteristics of the synthesized CdS NPs were investigated using the following techniques: FTIR, SEM, EDX, TEM, Ultraviolet–Visible spectroscopy, XRD, and Raman spectroscopy.

#### 4.2.1. Fourier Transform Infrared (FTIR) Spectroscopy

A FTIR spectrum of the CdS NPs was obtained using a Nicolet iS50 FTIR Spectrometer (Thermo Fisher Scientific, WI, USA). The CdS NPs sample was mounted on the sample holder and secured with clips before subjecting it to 16 scans within the range of 4000 to 400 cm^−1^ in absorbance mode at a resolution of 4 cm^−1^ [71].

#### 4.2.2. Scanning Electron Microscopic (SEM) and Energy-Dispersive X-Ray Spectroscopic (EDX) Analysis

The morphology of the synthesized nanoparticles was studied using scanning electronmicroscope (Vega 3 Model No. 2011, TESCAN, Brno, Czech Republic) in the secondary electron (SE) detector mode at an accelerating voltage of 20 kV. An energy-dispersive X-ray spectrometer (Oxford Instruments, X-Max, Abingdon, UK) fitted to the SEM was utilized to determine the purity and elemental composition of the synthesized CdS NPs. Sample preparation was performed by depositing a thin layer of the sample on a carbon tape attached to a copper stud, followed by carbon coating before examination.

#### 4.2.3. Transmission Electron Microscopy (TEM)

A JEOL transmission electron microscope (model JEM-2100, Tokyo, Japan) operating at 200 kV accelerating voltage and a beam current of about 107 µA was employed to study the structural and surface morphology. Sample preparation was performed by subjecting 3 mg of CdS NPs in 1 mL of absolute ethanol to ultrasonication for 30 min. For TEM analysis, a drop of CdS NP suspension was applied to a holey carbon-coated 300 mesh (3 mm) copper grid mounted on a filter paper, and then allowed to air-dry before being subjected to TEM analysis.

#### 4.2.4. UV-Vis Spectrophotometric Analysis

Optical properties of the nanomaterial were investigated with a UV-Visible spectrophotometer (Evolution 220; Thermo Fisher Scientific, WI, USA) within the wavelength range of 200–800 nm. Prior to analysis, 1 mg of CdS nanoparticles were dissolved in 2 mL of Milli-Q water and sonicated for 30 min to ensure uniform dispersion. The blank was read first to account for loss of light waves due to light scattering or absorption [60]. The cuvette was rinsed twice with a diluted portion of the prepared sample before being filled to about three-quarters of its volume with the prepared suspension. To prevent disturbances from ambient light, analysis was conducted under a covering [60].

#### 4.2.5. X-Ray Powder Diffraction (XRD)

The crystal phase of the nanoparticles was studied using a Malvern Panalytical Empyrean X-ray diffractometer (Malvern Pananalytical, Almelo, The Netherlands) with Cu-Kα radiation (λ = 1.54060 Å) under a voltage of 40 kV and 40 mA. Phase identification was performed using X’Pert HighScore Plus software (version 2.0), while crystal sizes were calculated using the Debye–Scherrer equation [72].

#### 4.2.6. Raman Spectroscopy

Raman measurements were performed at room temperature with LabRAM HR Evolution spectrometer (Horiba Scientific, Villeneuve-d’Ascq, France) using 514.5 nm excitation line from a mixed Ar/Kr ion laser in a backscattering geometry [73].

### 4.3. Antimicrobial Activity of Cadmium Sulfide Nanoparticles

Agar well diffusion [74], minimum inhibitory concentration (MIC) test [75], and minimum bactericidal concentration (MBC) [76] techniques were used to determine the antimicrobial activity of CdS NPs, in a concentration-dependent manner (50 mg/mL, 25 mg/mL, and 10 mg/mL) against two bacterial species: *Escherichia coli* (ATCC 25922) and *Staphylococcus aureus* (ATCC 25923) obtained from the Microbiology Laboratory, Vaal University of Technology, South Africa. The microorganisms were grown in Mueller–Hinton Broth (Biolab Merck, Darmstadt, Germany) at 37 °C for 24 h in a rotary shaker at 160 rpm after inoculating the broth with a loopful of bacteria.

#### 4.3.1. Agar Well Diffusion Technique

About 100 μL of overnight culture of the test microorganism was spread uniformly on Mueller–Hinton (Biolab Merck, Darmstadt, Germany) agar plates using a sterile disposable cotton swab [77]. Afterwards, wells of 5 to 6 mm in diameter were created aseptically onto the agar plates using the head of sterile 200 μL micropipette tips [60]. An amount of 50 μL of the CdS NPs sample was loaded into the respective wells. Neomycin (50 mg/mL) and distilled water were used as positive and negative controls, respectively. The test was performed in duplicate. The plates were incubated at 37 °C and room temperature (23 to 27 °C) for 24 h. The sensitivities of the test organisms to the samples were indicated by a clear zone (zone of inhibition) around wells [60].

#### 4.3.2. Minimum Inhibitory Concentration Assay

Minimum inhibitory concentration assay was used to determine the inhibitory effect of CdS NPs on the test organisms at different concentrations (50 mg/mL, 25 mg/mL and 10 mg/mL). Disposable microtitration 96-well plates were used for the tests. Test organisms, *Escherichia coli* and *Staphylococcus aureus*, were grown in MH broth on a shaker incubator at 150 rpm for 24 h at 37 °C. About 100 µL of the overnight-grown culture was added to the wells. This was followed by adding samples: CdS NPs, Neomycin, and distilled water on the first row of the microtitration plates, in duplicates. A two-fold dilution of the samples followed. The microplates were incubated at 37 °C and room temperature (23 to 27 °C) for 24 h. Following this, 30 µL of 0.02% resazurin dye was added to all the wells and incubated again at the same temperatures. After 24 h, the wells were inspected for color change, from blue to pink.

#### 4.3.3. Minimum Bactericidal Concentration (MBC)

MBC endpoint is defined as the state when >99.9% of the bacterial population is killed at the lowest concentration of an antimicrobial agent and can be assessed by examining the number of surviving cells (CFU/mL) on Nutrient Agar (NA) plates [78]. The MBC was determined using the MIC 96-well microplate results. From each microplate, aliquots of 50 µL were taken from wells below the MIC, at the MIC, and above the MIC. These aliquots were aseptically spread onto Nutrient Agar plates and incubated for 24 h at 37 °C and at room temperature (23–27 °C). Following incubation, plates were examined for bacterial growth to identify the lowest concentration that produced no visible colonies, which was taken and recorded as the MBC [76,78].

## 5. Conclusions

This study successfully synthesized and characterized CdS NPs and further investigated their antimicrobial properties. FTIR studies revealed successful synthesis of CdS NPs through the Cd–S stretching bond. Raman spectroscopy analysis suggested that the nanoparticles possessed first- and second-order longitudinal optical phonon vibrations at 302.3 cm^−1^ and 601 cm^−1^. SEM and TEM images showed that CdS NPs are fine, near-spherical, and crystalline-like in structure with an average particle size of 7.236 nm, while the EDX confirmed that the synthesized nanoparticles are pure. The Uv-Vis absorption spectrum showed light absorbance in the visible light region. The X-ray diffraction suggested the existence of a cubic blend of CdS as specified by the JCPDS indexing card. Antimicrobial assays showed that CdS NPs have antimicrobial activity against the tested organisms. Antimicrobial activity of CdS NPs was seen as dose-dependent; the higher the concentration of CdS NPs, the more effective the nanoparticles. CdS NPs were more effective against Gram-negative *Escherichia coli* and showed higher bactericidal activity at 37 °C and higher bacteriostatic activity at room temperature (23 to 27 °C). Cadmium sulfide nanoparticles are potential new-generation antimicrobial agents and could serve as a good alternative to conventional antimicrobials, which are becoming less effective due to increasing pathogen resistance.

## Figures and Tables

**Table 1:** Diameters of zones of inhibition by cadmium sulfide nanoparticles.

Mean Zones of Inhibition (in mm)								
Microorganisms	CdS NPs (mg/mL)						Neomycin (mg/mL)	
	50		25		10			
	RT ^1^	37 °C	RT	37 °C	RT	37 °C	RT	37 °C
*E. coli*	20 ± 1.53	16 ± 1	16 ± 2.31	13 ± 2	12 ± 0.58	12 ± 0.58	24 ± 5.29	27± 1.53
*S. aureus*	24 ± 2.31	14 ± 3.22	19 ± 1.16	10 ± 2.52	17 ± 0	9 ± 2.52	27 ± 2.31	25 ± 0

^1^ Room temperature (23 to 27 °C).

**Table 2:** Minimum inhibitory/bactericidal concentration values of cadmium sulfide nanoparticles.

Minimum Inhibitory/Bactericidal Concentrations									
Microorganisms		CdS NPs (mg/mL)						Neomycin (mg/mL)	
		50		25		10			
		37 °C	RT ^1^	37 °C	RT	37 °C	RT	37 °C	RT
*E. coli*	MIC ^2^	6.25	3.125	12.5	6.25	25	12.5	˂0.391	˂0.391
	MBC ^3^	6.25	12.5	12.5	25	50	- ^4^	˂0.391	˂0.391
*S. aureus*	MIC	6.25	6.25	12.5	6.25	-	25	˂0.391	˂0.391
	MBC	12.5	-	25	-	-	-	˂0.391	˂0.391

^1^ Room temperature (23 to 27 °C); ^2^ minimum inhibitory concentrations; ^3^ minimum bactericidal concentrations, ^4^ absence of antimicrobial activity.

## Data Availability

The original contributions presented in this study are included in the article. Further inquiries can be directed to the corresponding authors.

## Abbreviations

The following abbreviations are used in this manuscript:

CdS NPs	Cadmium sulfide nanoparticles
rt	Room temperature
XRD	X-ray diffraction
MIC	Minimum inhibitory concentration
MBC	Minimum bactericidal concentration
ROS	Reactive oxygen species
DNA	deoxyribonucleic acid
FTIR	Fourier transform infrared
EDX	Energy-dispersive X-ray
SEM	Scanning electron microscopy
TEM	Transmission electron microscopy
SDGs	Sustainable Development Goal
MH	Mueller–Hinton agar
JCPDS	Joint Committee on Powder Diffraction Standards
